# Reversible Silencing of Cytomegalovirus Genomes by Type I Interferon Governs Virus Latency

**DOI:** 10.1371/journal.ppat.1003962

**Published:** 2014-02-20

**Authors:** Franziska Dağ, Lars Dölken, Julia Holzki, Anja Drabig, Adrien Weingärtner, Johannes Schwerk, Stefan Lienenklaus, Ianina Conte, Robert Geffers, Colin Davenport, Ulfert Rand, Mario Köster, Siegfried Weiß, Barbara Adler, Dagmar Wirth, Martin Messerle, Hansjörg Hauser, Luka Čičin-Šain

**Affiliations:** 1 Department for Vaccinology/Immune Aging and Chronic Infection, Helmholtz Centre for Infection Research, Braunschweig, Germany; 2 Department of Medicine, University of Cambridge, Cambridge, United Kingdom; 3 Department of Gene Regulation and Differentiation, Helmholtz Centre for Infection Research, Braunschweig, Germany; 4 Department of Molecular Immunology, Helmholtz Centre for Infection Research, Braunschweig, Germany; 5 Department for Genome Analytics, Helmholtz Centre for Infection Research, Braunschweig, Germany; 6 Department of Pediatric Pneumology and Neonatology, Medical School Hannover, Hannover, Germany; 7 Department of Virology, Max von Pettenkofer Institute, Ludwig Maximillian University, Munich, Germany; 8 Department for Virology, Medical School Hannover, Hannover, Germany; University of Arizona, United States of America

## Abstract

Herpesviruses establish a lifelong latent infection posing the risk for virus reactivation and disease. In cytomegalovirus infection, expression of the major immediate early (IE) genes is a critical checkpoint, driving the lytic replication cycle upon primary infection or reactivation from latency. While it is known that type I interferon (IFN) limits lytic CMV replication, its role in latency and reactivation has not been explored. In the model of mouse CMV infection, we show here that IFNβ blocks mouse CMV replication at the level of IE transcription in IFN-responding endothelial cells and fibroblasts. The IFN-mediated inhibition of IE genes was entirely reversible, arguing that the IFN-effect may be consistent with viral latency. Importantly, the response to IFNβ is stochastic, and MCMV IE transcription and replication were repressed only in IFN-responsive cells, while the IFN-unresponsive cells remained permissive for lytic MCMV infection. IFN blocked the viral lytic replication cycle by upregulating the nuclear domain 10 (ND10) components, PML, Sp100 and Daxx, and their knockdown by shRNA rescued viral replication in the presence of IFNβ. Finally, IFNβ prevented MCMV reactivation from endothelial cells derived from latently infected mice, validating our results in a biologically relevant setting. Therefore, our data do not only define for the first time the molecular mechanism of IFN-mediated control of CMV infection, but also indicate that the reversible inhibition of the virus lytic cycle by IFNβ is consistent with the establishment of CMV latency.

## Author Summary

Cytomegalovirus (CMV) is a widespread herpesvirus that establishes a détente with the host immune system. Therefore, the CMV reactivates from latency in immunocompromised hosts, resulting in life-threatening disease of the vulnerable patients. However, the exact mechanism by which the immune system keeps CMV at bay remains incompletely understood. To address this question, we have used a reporter system, based on infection of cells with the mouse CMV. Our results showed that interferon (IFN), a well-known antiviral protein, blocks CMV replication at the earliest stages after the virus has entered the cell. More importantly, removing the IFN from the infected cells restarted MCMV replication, indicating that its effects are consistent with viral latency. We showed that IFN blocked virus replication by inducing the expression of proteins located in the nuclear domain 10 (ND10), a compartment in the nucleus of cells to which the incoming viral genomes are directed. Similarly, IFN was sufficient to block CMV reactivation from cells of latently infected mice. In conclusion, IFN had the ability to drive CMV into a quiescent state matching the formal definition of latency and was sufficient to prevent reactivation of bona fide latent CMV.

## Introduction

Herpesviruses are characterized by their ability to establish a lifelong latent infection in their natural host and reactivate upon immunosuppression. Cytomegaloviruses (CMV) are paradigmatic β-herpesviruses, characterized by strict species specificity, but highly prevalent in numerous mammalian species [1]. Human CMV (HCMV) prevalence ranges from 30 to 90% [2]. While primary infection and latency are usually asymptomatic in immunocompetent individuals, immune suppression results in virus reactivation, which is associated with substantial morbidity and mortality. In particular, CMV reactivation may result in allograft rejection, pneumonia or gastroenteritis in recipients of solid-organ and bone-marrow transplants [3]. Understanding the molecular mechanisms involved in the establishment and maintenance of latency is fundamental for developing effective countermeasures to CMV disease in high-risk populations.

The human and the murine CMV (MCMV) share many biological properties. As such, MCMV infection of mice is a widely used *in vivo* model of CMV immunity and latency. Latency is characterized by the persistence of silenced virus genomes in the absence of infectious viral particles [4]. Both HCMV and MCMV infect a variety of cell types in their respective host [5], [6], but latency seems to be confined to distinct cell types, such as cells of the myeloid lineage [5], [7]–[10]. While HCMV latency in endothelial cells remains controversial [11], [12], strong evidence supports the notion that liver sinusoidal endothelial cells (LSECs) are a site of MCMV latency [13].

Similar to HCMV, chromatinization and recruitment of cellular repressors to the viral DNA and to the major immediate early (MIE) gene locus are critically involved in the *in vivo* establishment of MCMV latency [14], [15]. The IE genes regulated by the MIE promoter (MIEP) encode the first viral proteins expressed during productive infection, and act as essential transactivators of early and late genes [16]. Reactivation of latent HCMV from *in vivo* infected myeloid progenitor cells was shown to be related to MIE chromatinization [17]. Namely, the latent viral genome is associated with repressive chromatin in immature myeloid cells, whereas virus reactivation is accompanied by chromatin remodeling and initiation of transcription at the MIE locus during cell-differentiation. Therefore, MIEP transcriptional activity is generally considered an important checkpoint in CMV latency and reactivation.

In the immunocompetent host, primary infection is controlled by a combination of immunological effectors. Infected cells are directly eliminated, e.g. by cytotoxic effects of NK or T-cells. In addition, the spread of infectious virus is restricted by antibodies or by cytokines that reduce the permissiveness of cells for viral replication. Cytokines such as type I (IFNα/β) or type II interferons (IFNγ) are critical in the control of acute infection [18], [19]. They exert their antiviral action by activating immune effector cells like DCs, T cells or NK cells, but also by inducing transcriptional programs which suppress virus replication in target cells [20].

While it is generally accepted that interferons limit virus spread without killing the infected cell, the exact mechanism of their antiviral action remains unclear. Most importantly, it remains unclear if their effect results in CMV clearance, or if the viral replication is merely suppressed while genomes are maintained in the infected cell. A reversible block of viral replication prior to immediate-early expression would argue that interferons play a key role in the establishment of CMV latency. In a seminal paper, Presti et al. showed that mice that lack type II IFN receptors maintain a productive MCMV infection and that MCMV reactivation from explants of latently infected mice may not be observed in the presence of IFNγ [19]. Unfortunately, this experimental setting could not differentiate if the IFNγ truly suppresses virus reactivation by acting directly in latent cells or merely inhibited viral spread to other cells upon reactivation. In contrast, the role of type I IFN in the establishment and maintenance of latency is difficult to investigate *in vivo*, as IFNα/β receptor knockout (IFNAR^−^) mice are about 1000-folds more susceptible to MCMV than wild-type mice, and die within a few days post infection [19]. Nevertheless, in vitro experiments showed that IFNβ induced by lymphotoxin α reversibly suppresses HCMV and MCMV gene expression and replication [21]. Moreover, MCMV replication in macrophages is transiently suppressed by synergic action of IFNγ and type I interferons [22]. However, both publications showed that the suppression was only partial, because viral gene expression was reduced, but still detectable [21], [22]. Therefore, the effects of this axis seemed to reflect simmering lytic replication, rather than bona fide viral latency.

In this study, we show that MCMV replication may be completely, but reversibly, inhibited in cells that respond to IFNβ, in a manner consistent with viral latency. On the other hand, cells which failed to respond to IFNβ were permissive for MCMV replication. We show that the inhibition of MCMV replication by IFNβ depends on the inhibition of viral gene expression at the level of IE transcription mediated by nuclear domain 10 (ND10) components, which is fully reversible even after extended culture of *in vitro* infected cells and in cultures of endothelial cells derived from latently infected mice. In summary, our data indicate that reversible silencing of viral genomes by IFN-induced ND10 components is a key contributor to the establishment of CMV latency.

## Results

### Restriction of MCMV Replication by IFNβ

**Figure 1:**
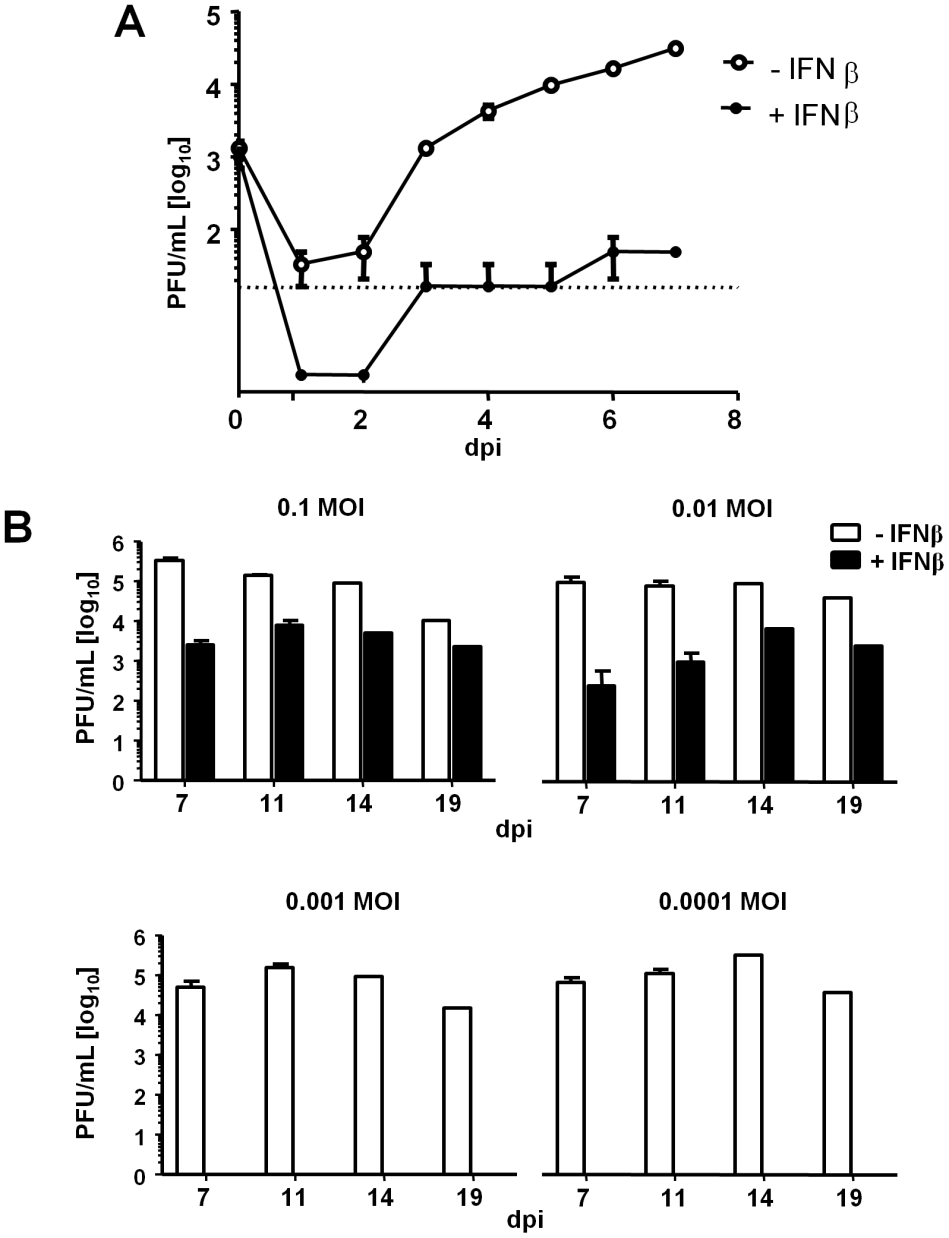
IFNβ restricts MCMV replication in LSECs. LSECs were incubated for 24(+IFNβ, 500 U/mL) or without IFNβ (−IFNβ), and infected with MCMV. The same medium was added following infection. (**A**) Supernatants (SN) from cells infected at an MOI 0.001 were collected daily up to 7 dpi and titrated on IFNAR^−/−^ MEFs. The average titers (PFU/mL) from triplicates are shown and error bars indicate SD. (**B**) LSECs were MCMV infected at indicated MOIs and ¼ of the SN was harvested for titration at 7, 11, 14 and 19 dpi, and substituted with medium ± IFNβ. Titration was performed on IFNAR^−/−^ MEFs. Histograms show average titers (PFU/mL) from replicates ± SD.

LSECs are a site of MCMV latency [13]. We recently described an LSEC line which enters cell cycle in a doxycycline-dependent manner and is highly permissive for MCMV infection [23]. To study type I interferon (IFN) effects on MCMV replication in quiescent LSECs, growth-arrested cells were incubated with IFNβ for 24 h, infected with MCMV at a multiplicity of infection (MOI) of 0.001 and viral growth was assayed for a week. Until 5 days post infection (dpi), infectious virus was only exceptionally detected in supernatants (SN) of IFNβ-treated LSECs, and viral titers were substantially diminished on 6 and 7 dpi, as compared to untreated cells (Figure 1A). Therefore, consistent with previous reports, IFNβ treatment resulted in delayed viral growth and reduced viral titers, but did not completely block MCMV replication.

**Figure 2:**
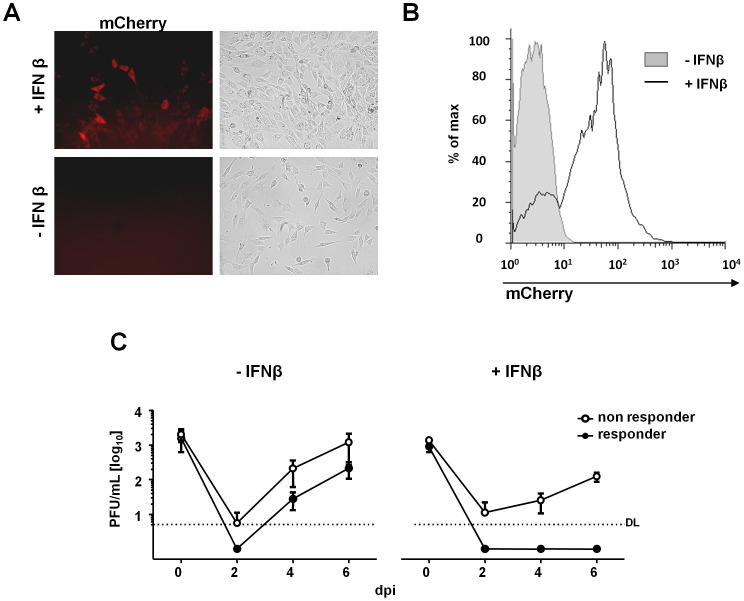
MCMV replication is completely blocked in IFNβ-responsive cells. NIH3T3 cells expressing the IRF7–mCherry fusion protein were treated with 500 U/mL IFNβ for 24 h and analyzed for mCherry expression by both fluorescence microscopy and flow cytometry. Representative microscopic pictures (**A**) and flow cytometry (**B**) of IFNβ-treated (white histogram) and untreated (grey histogram) cells are shown. (**C**) IRF7 reporter cells were cultured in the presence of IFNβ for 24 h and FACS sorted as responder (mCherry^+^) or non-responder (mCherry^−^) cells. Both cell populations were cultivated in absence (left), or presence of IFNβ (right), infected with MCMV Δm157 eGFP at 0.01 MOI. SN were collected at 0 (input virus), 2, 4 and 6 days post infection (dpi). Virus titrations were performed on IFNAR^−/−^ MEFs. Graphs show average titers (PFU/mL) of three independent experiments ± SEM.

Recently, we reported that a minor proportion of cells remain unresponsive even to high doses of type I interferon [24]. We speculated that MCMV infection of IFNβ-unresponsive cells may generate sufficient amounts of virus to overcome the barrier to infection installed by IFNβ pre-treatment. In that case, infection at low MOI would increase the chance that exclusively IFN-responsive cells are infected and that the infection becomes fully contained. We thus repeated the initial experiment with reduced doses of MCMV, up to a dilution of 1 plaque forming unit (PFU) per 10,000 cells, and monitored the long-term IFNβ effects for up to 19 dpi in growth-arrested LSECs (20,000 cells/well). While IFNβ treated samples infected at an MOI of 0.1 and 0.01 showed substantial virus titers by 7 dpi and later (Fig. 1B, upper panels), MCMV replication was completely abrogated, when the infection was performed at MOIs below 0.01 (Fig. 1B, lower panels). It is important to note that both an MOI of 0.001 and 0.0001 still resulted in complete cell lysis and high viral titers in IFNβ-naïve samples (Fig. 1B, lower panels, white bars). These findings were consistent with the model that CMV infection is contained at very low MOIs because it is restricted to IFNβ-responsive cells. To confirm this hypothesis, we used reporter cells that express an IRF-7-mCherry fusion protein under the control of the IFNβ-responsive IRF-7 promoter (Figure 2A). Reporter cell stimulation with IFNβ (500 U/ml) revealed a small but notable population of non-responding cells (Figure 2B). We separated the cells into responders and non-responders by fluorescence activated cell-sorting (FACS) and then infected them with a dose (MOI 0.01), which could not be contained by IFNβ in the previous experiment (Figure 1B). In the absence of further IFNβ treatment, MCMV titers were diminished in cells which responded to IFNβ (Figure 2C, left diagram). More importantly, virus replication was completely abolished upon continuous IFNβ treatment, but only in IFNβ-responsive cells (Figure 2C, right diagram). In summary, these experiments demonstrate that IFNβ pretreatment is sufficient to restrict MCMV replication in cells which respond to IFNβ. However, virus expansion in the few IFN-unresponsive cells eventually overcomes the resistance of the IFNβ-responsive population.

### IFNβ Silencing of MCMV Immediate Early Gene Expression Is Reversible

**Figure 3:**
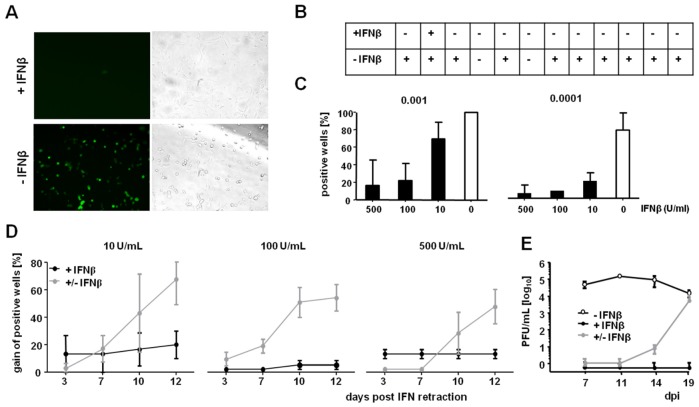
Inhibition of viral replication by IFNβ is reversible and occurs prior to immediate-early gene expression. LSECs were treated with IFNβ for 24 h and infected with MCMV^r^. (**A**) Representative EYFP fluorescence microscopy of IFNβ treated (500 U/mL) or untreated LSECs at 7 dpi; MOI = 0.0001 is shown. (**B**) Twelve wells per condition were analyzed at multiple time points of infection. Wells containing as little as a single infected cell were classified as positive. (**C**) Cells were treated with 10, 100 or 500 U/mL of IFNβ for 24 h, infected at the indicated MOIs and the percentage of positive wells (of 12) was determined at 7 dpi. Histograms indicate average values from three independent experiments, error bars show SD. (**D**) IFNβ-treated LSECs were infected with an MOI of 0.0001. At dpi 7, IFNβ was removed in selected wells (+/− IFNβ) and wells were monitored for EYFP expression at the indicated time points post IFN retraction. The gain of positive wells as mean percentage from three independent experiments ± SEM is shown. (**E**) LSECs were pre-stimulated with 500 U/mL IFNβ and infected with 0.001 MOI of MCMV^r^. SN were collected on 7, 11, 14 and 19 dpi and titrated on MEFs. Graphs show average titers (PFU/mL) from duplicates (± SD). See also Figure S1.

IFNβ abrogated productive MCMV replication in LSECs at low MOIs. To elucidate at which step of infection this block was exerted, we infected LSECs with a recombinant MCMV that expresses two different fluorescent proteins as reporters that reflect the activity of the MCMV major immediate early promoter (MIEP). This virus was named MCMV^r^ and contains an ectopically inserted, full-length MIEP sequence flanked by the yellow fluorescent protein EYFP, driven by the ie1/3 promoter, and the red fluorescent protein tdTomato that is controlled by the ie2 promoter. MCMV^r^ grows like WT MCMV *in vitro* and expresses EYFP and tdTomato with the same scale and kinetics like the MCMV IE1 and IE2 genes, respectively [23]. To determine the onset of viral infection we monitored EYFP expression, which occurs earlier than tdTomato [23], in line with reports that the ie1 gene is immediately expressed at high levels during primary lytic infection and reactivation [25], [26]. MCMV^r^ infection of LSECs resulted in strong EYFP-expression, which was hardly detectable in IFNβ-treated cells (Figure 3A). To determine if IFNβ completely abrogated MCMV IE gene expression, LSECs were infected with MCMV^r^ in 96-well plates, scanned for reporter gene expression, and wells were classified as positive when even a single EYFP-fluorescent cell could be observed within a week of infection (representative result of an experiment in 12 wells per condition is shown in Figure 3B). Different IFNβ concentrations were tested with various MOIs, to assess the conditions that allow the complete suppression of viral genes, and the percentage of positive wells was defined (Figure 3C). 500 and 100 U/mL of IFNβ blocked all viral gene expression in more than 80% of wells at 0.001 and 0.0001 MOI for 7 dpi, whereas 10 U/mL showed similar activity only at the lower MOI (Figure 3C). Importantly, in wells that showed one single positive cell, the progress of the lytic infection was irreversible and the virus would always spread to nearby cells. We next tested if the suppressive effect of IFNβ on MCMV gene expression is permanent or reversible, by removing IFNβ at 7 dpi and monitoring the cells for additional 12 days. Remarkably, IFNβ removal resulted in viral gene expression (Figure 3D) and production of infectious virus (Figure 3E) about 7–10 days later, while the percentage of EYFP positive wells remained unchanged (Figure 3D) and viral titers undetectable (Figure 3E) in wells permanently treated with IFNβ. We concluded that IFNβ reversibly suppresses MCMV replication before or at the time of MIEP-driven gene expression. Of note, the same finding was observed following infection with the γ-herpesvirus MHV68 (Figure S1B). In contrast, vesicular stomatitis virus (VSV), which could be efficiently suppressed with IFNβ, was not able to replicate after IFNβ-retraction (Figure S1A). In conclusion, IFNβ silenced the replication of three different viruses. However, this was only reversible for infections with the two herpesviruses.

### IFNβ Acts at the Level of MIEP-Driven Gene Expression

**Figure 4:**
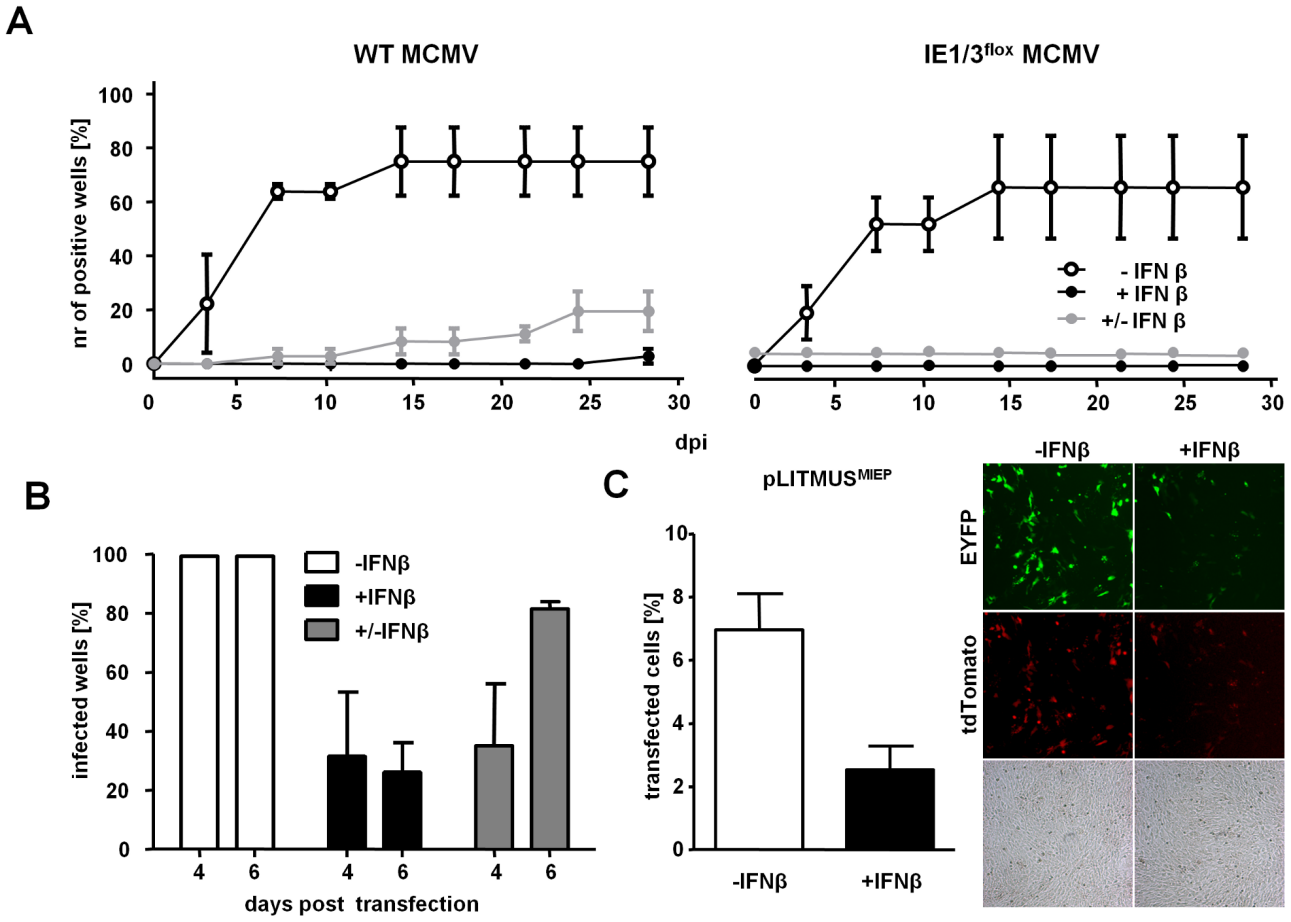
IFNβ suppresses MCMV immediate early gene expression and not virus entry. (**A**) MEF expressing Cre recombinase were treated with IFNβ and infected with WT or IE1/3^flox^ MCMV (0.0001 MOI). At 7 dpi, IFNβ was removed in selected wells (± IFNβ) or resupplied at regular intervals until day 28 (+IFNβ). Control cells were infected in the absence of IFNβ (−IFNβ). The mean percentage of wells showing viral plaques ± SEM at indicated time points from three independent experiments is shown. (**B**) MEFs were transfected with the MCMV^r^ BAC and supplied with IFNγ 5 h later. After 4 days, the wells were inspected for signs of EYFP expression and wells were either resupplied with IFNβ (+IFNβ) or, in selected wells, it was removed from the cell medium (± IFNβ). Wells were reassessed for EYFP-expression at 6 days post transfection. The percentage of positive wells showing EYFP-expression from two independent experiments (± SD) is shown. (**C**) NIH3T3 fibroblasts were transfected with a plasmid expressing EYFP and tdTomato driven by the MCMV MIEP promoter. The cells were treated with IFNβ at 5 h post transfection and analyzed by fluorescence microscopy and flow cytometry at 3 days post transfection. Representative fluorescent images are shown and histograms indicate the percentage of transfected cells (EYFP+tdTomato) from triplicates (± SD). See also Figure S2.

IFNβ reversibly inhibited MCMV replication and expression of genes driven by the ie1/3 promoter. This could be due to a direct block of IE-gene expression, or effects that occurred during the viral entry into the cells. To test if the reversible suppression by IFNβ occurs after the virus has entered the cell and the viral genomes are delivered to the nucleus, we generated a recombinant MCMV in which the ie1/3 locus is flanked by two loxP sites (IE1/3^flox^ MCMV) which results in IE1 and IE3 deletion when the genome is recognized by the Cre recombinase in the cell nucleus (Figure S2A). Importantly, IE1/3^flox^ MCMV replicates in Cre-expressing cells, probably due to rapid MIEP-driven gene expression, which precedes the Cre-mediated deletion of target sites (Figure 4A and Figure S2B). We considered that the Cre recombinase would have sufficient time to excise the IE1 and IE3 genes and abrogate reactivation upon IFNβ retraction, if IFNβ blocked viral gene expression after genome delivery to the nucleus. Cre-expressing MEFs were pre-treated with IFNβ, infected with IE1/3^flox^ MCMV or WT MCMV and the wells were scanned for viral plaques. Viral replication of WT and IE1/3^flox^ MCMV was efficiently blocked in cells which constantly received IFNβ over a time period of 4 weeks (Figure 4A). IFNβ removal at 7 dpi resulted in virus replication in several wells infected with WT MCMV, consistent with the data obtained from MCMV^r^-infected LSECs (Figure 3D). In contrast, IE1/3^flox^ MCMV failed to replicate upon IFNβ removal from Cre-MEFs, indicating that the IE1/3^flox^ MCMV genomes were exposed to Cre-recombinase in the nucleus, and the deletion of the ie1/3 genes abrogated the ability of the virus to replicate upon IFNβ-removal.

Since our data indicated that MCMV genomes are delivered to the cell nucleus in the presence of IFNβ, we assumed that it directly inhibits viral gene expression. To test the ability of IFNβ to impair viral gene expression in absence of virion components, we delivered the MCMV genomes into cells by transfection [27]. Since transfection is less efficient in LSECs than in MEFs (data not shown), we transfected MEFs with the MCMV^r^ bacterial artificial chromosome (BAC) and treated them with IFNβ immediately upon transfection. MIEP-driven expression was detected by fluorescence microscopy for EYFP expression. Four days post transfection, EYFP was observed in all wells transfected in the absence of IFNβ treatment. In contrast, EYFP could be observed in only few of the IFNβ-treated wells (Figure 4B). Most importantly, removing IFNβ resulted in the restoration of MIEP activity by day 6 (Figure 4B). To understand if the inhibition of gene expression was exclusive to the MIEP promoter, or to any incoming DNA, fibroblasts were transfected with plasmids expressing the EYFP and tdTomato under the control of the MCMV MIEP or with plasmids expressing reporter genes under the control of other promoters (SV40 and HCMV). The expression of all reporter genes was substantially diminished in IFNβ treated cells (Figure 4C and data not shown), indicating that IFNβ suppresses gene expression in a manner that is not specific for the MCMV MIEP but to any incoming DNA. Together, these data provide strong evidence that IFNβ-mediated reversible suppression of viral replication occurs directly at the level of gene transcription of foreign DNA entering the nucleus.

### The Suppression of Immediate Early Gene Expression by IFNβ Is Due to Transcriptional Silencing Mediated by ND10 Components

**Figure 5:**
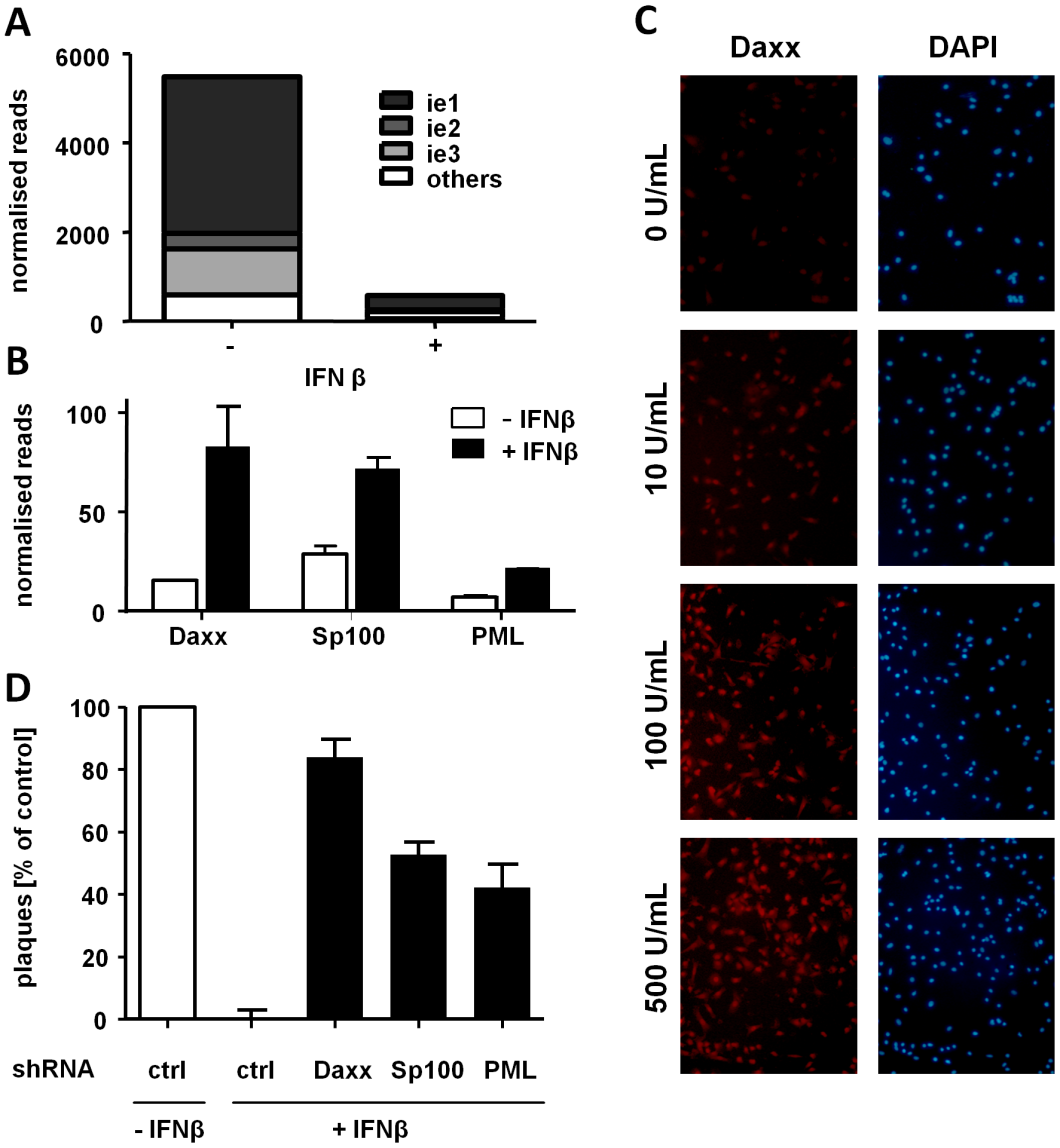
IFNβ suppresses MCMV IE gene expression at the transcriptional level by inducing ND10 genes. LSECs were treated with medium ± IFNβ (500 U/mL) and 24 h later infected with MCMV WT. Virus absorption was restricted to 5 minutes to improve time resolution. Nascent RNA samples were collected at 1 hpi and used for deep-sequencing. (**A**) Histograms show normalized reads of viral transcripts in IFNβ-treated and untreated LSECs from two replicates. The fraction of the viral transcriptome corresponding to ie1, ie2 or ie3 transcripts is indicated (**B**) Counts of normalized Daxx, Sp100 and PML transcripts in IFNβ-treated and non-treated LSECs. Histograms show normalized reads from two replicates (**C**) LSECs were treated with 10, 100 or 500 U/mL IFNβ or left untreated for 24 h and then stained for Daxx. Representative fluorescent pictures are shown. (**D**) LSECs were transfected with plasmids expressing shRNA against Daxx, Sp100 or PML, treated with IFNβ or left untreated and infected 24 h later with 0.01 MOI of MCMV^r^. Control cells were transfected with scrambled shRNA in the presence or absence of IFNβ and infected as above. Viral plaques were counted 4 dpi, normalized to represent IFNβ-untreated samples as 100 and average normalized PFU from three independent experiments ± SD are shown. See also Figure S3 and Table S1, S2 and S3.

To formally show that IFNβ blocks MCMV replication at the level of gene transcription rather than translation, we analyzed the viral transcriptome of IFNβ-treated LSECs immediately upon MCMV infection. CMV particles carry significant amounts of virion-associated RNA [28], which, upon delivery to infected cells, impede the detection of *de novo* synthesized immediate early and early viral transcripts. We therefore metabolically labeled newly transcribed RNA with 4-thiouridine (4sU), isolated the labeled RNA by thiol-specific biotinylation and streptavidin-precipitation [29], and deep-sequenced the newly transcribed RNA. To observe the effect of IFNβ at the earliest possible time point after the infection, we adapted the infection protocol and incubated the cells with infectious virus for 5 min only, using an infectious dose that was normalized to match an MOI of 10 in standard infection and virus absorption. This allowed us to focus our analysis on viral transcripts generated during the first hour of infection (hpi). At 1 hpi, the IE gene transcripts were detectable and comprised the majority of viral transcripts, whereas they were highly diminished in IFNβ-treated LSECs (Figure 5A and Table S1). It is important to note that IFNβ treatment also diminished all other viral transcripts that could be detected at 1 hpi, consistent with the observed global suppression of reporter gene expression in all tested expression plasmids. Thus, IFNβ acts at the level of MCMV gene transcription, resulting in strong transcriptional repression of all viral genes expressed in the first hour of infection.

Since IFNβ inhibited MCMV replication at the level of viral IE gene transcription we hypothesized that this effect might be mediated by induction of nuclear domain 10 (ND10) components. ND10 bodies are nuclear structures known to associate with incoming viral DNA restricting CMV replication [30]–[32]. Hence, we screened the host-cell transcriptome for members of the ND10 and compared their transcriptional level in untreated and IFNβ-treated LSECs. Interestingly, three major components of the ND10: Daxx, Sp100 and PML, were upregulated in IFNβ-treated LSECs (Figure 5B and Table S2), consistent with published data [33], [34]. This was confirmed by immunofluorescence staining for Daxx (Figure 5C) and RT-PCR for all three components (Figure S3). To define the relevance of these factors in the IFNβ-mediated suppression of MCMV replication, we performed shRNA-mediated knockdowns of these three ND10 components, which reduced their mRNA levels to those seen in IFN-untreated cells (Figure S3). More importantly, each of the three knockdowns was sufficient to almost completely restore MCMV replication in the presence of IFNβ (Figure 5D). Collectively, these data highlight a key role of ND10 bodies in the transcriptional silencing of CMV gene expression induced by IFNβ.

### IFNβ Induces a Reversible Silencing of MCMV *In Vivo*

**Figure 6:**
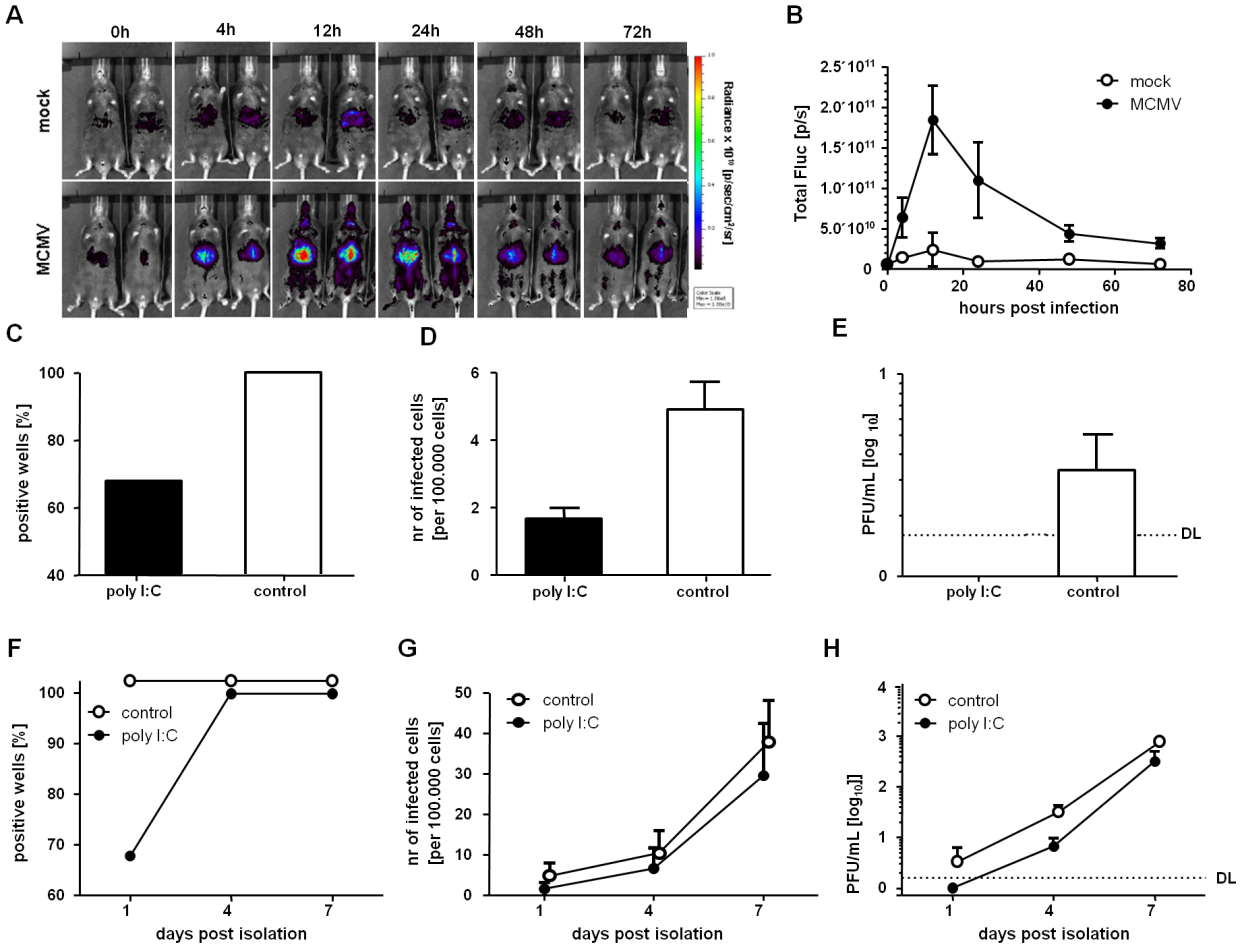
IFNβ stimulation *in vivo* induce a reversible silencing of MCMV in LSECs. (A) Whole-body *in vivo* imaging of luciferase activity upon intraperitoneal infection of Mx2Luc reporter mice with 1×10^6^ pfu MCMV WT. Control mice were mock infected with PBS. The rainbow scale depicts the strength of radiance expressed as photons per second per cm^2^ per steradian (sr). Imaging was performed at 4, 12, 24, 48 and 72 hpi. (B) Quantification of luciferase activity by region of interest (ROI) analysis of the liver. Two independent experiments were performed with similar results; one representative experiment is shown. Mean values of two mock- and three MCMV-infected mice are shown and error bars indicate SD (C, F) LSECs were isolated from MCMV^r^ infected IFNβ-reporter mice at 72 hpi and dispensed in a 96-well plate (28 replicates per condition). The wells were monitored for EYFP expression and the percentage of EYFP^+^ wells at day 1 post isolation (C) or the kinetic on days 1, 4 and 7 (F) is shown. (D, G) EYFP^+^ LSECs were counted and normalized to positive events per 100.000 cells. Mean values and SEM at day 1 (D) post isolation, or the kinetic on day 1, 4 and 7 (G) are shown. (E, H) Supernatants from isolated LSECs were collected and titrated on MEFs. The graph shows the mean and SEM of triplicates on day 1 post isolation (F) or the dynamic monitoring at days 1, 4 and 7 post isolation (G).

While our results showed very clear IFNβ effects on MCMV lytic replication, it remained open if it also induces MCMV latency *in vivo*. To understand how MCMV infection influences the production IFNβ *in vivo*, we infected transgenic mice which carry a luciferase reporter gene under the control of the MX2 promoter [35], a well-characterized IFN stimulated gene (ISG). Luciferase activity could be detected in the MCMV-infected mice already at 4 hpi (Figure 6A), indicating immediate production of IFN upon MCMV infection (Figure 6A). The reporter gene signal peaked at 12 hpi and declined thereafter, although a robust luciferase signal could still be detected by 72 hpi (Fig. 6A, 6B). Interestingly, the response to IFN was most prominent in the liver region throughout the time of monitoring.

To determine if *in vivo* IFNβ-stimulation also transiently silences MCMV in LSECs, we infected mice with MCMV^r^ in which the production of IFNβ was induced prior to infection. For this, we used a previously described IFN-β reporter mouse (IFN-β^+/Δβ-luc^), allowing the visualization of IFNβ expression by *in vivo* imaging using firefly luciferase as a reporter [36]. These mice were stimulated with poly I:C and a high activity of the IFNβ promoter could be detected 4 h after poly I:C injection but not in mock treated mice (Figure S4), consistent with the kinetic of MCMV infection (Fig. 6A). Mice were infected with MCMV^r^ at 8 h post stimulation, and LSECs were isolated from the liver of the infected mice at 72 hpi. LSECs were cultivated for 7 days and analyzed for reporter gene expression at 1, 4 and 7 days post isolation. After 1 day, MCMV reporter gene expression (EYFP) could be detected in all of the wells with LSECs that were isolated from control mice, infected in the absence of poly I:C. In contrast, MCMV ie gene expression was absent in about 1/3 of the wells containing LSECs from poly I:C-stimulated mice (Figure 6C). This was not merely a random redistribution of the EYFP+ cells to fewer wells, because the overall number of EYFP^+^ cells was substantially reduced in LSECs from poly I:C treated mice (Figure 6D). We considered the possibility that the absence of viral gene expression upon *in vivo* IFN induction is a result of a hindered viral entry in the LSECs. However, this scenario seemed unlikely, because we could not detect any infectious MCMV in the supernatants from poly I:C pre-treated LSEC (Figure 6E), while control LSECs showed detectable titers, probably as a result of ongoing virus shedding in the first 24 hours of culture. To understand if the *in vivo* MCMV suppression by IFN was due to reversible silencing of gene expression, the cells isolated from poly I:C-treated mice were further cultivated and MCMV gene expression was monitored at 4 and 7 days post isolation and EYFP expression could be observed in all of the wells, including those that were negative at 1 day post isolation (Figure 6F). Likewise, the number of EYFP^+^ cells increased upon cultivation, and by 4 and 7 days post isolation the LSECs from poly I:C treated mice showed similar levels as the controls (Figure 6G). Finally, this was accompanied by full virus reactivation, as demonstrated by the emergence of infectious virus in the supernatants at 4 days post isolation in the IFNβ-stimulated LSECs, and by its expansion by day 7 (Figure 6H). In conclusion, the infection of LSECs stimulated with IFNβ *in vivo* increased the proportion of cells that contained silent MCMV genomes that were able to re-initiate the replication cycle after explantation, upon several days of *ex vivo* cultivation. To confirm that this also occurs in the course of natural infection, in wild type mice and in absence of poly I:C treatment, we isolated LSECs from BALB/c mice at 72 hpi infection with MCMV^r^ and monitored EYFP expression on day 1 and 4 post isolation. By seeding LSECs at a lower concentration per well (50,000 cells per well, instead of 70,000), and using mice expressing IFNβ from both of its alleles (luciferase expression in reporter IFN-β^+/Δβ-luc^ mice is possible due to a monoallelic exclusion of IFNβ expression), we established conditions where MCMV IE gene expression was completely abrogated in absence of poly I:C prestimulation, because 7 out of 26 wells showed no EYFP expression at all on day 1 post isolation. The majority of these wells (5 out of 7) became positive for EYFP by day 4 post isolation (data not shown), demonstrating that viral genomes, but no gene expression, were present in some cells immediately upon infection. These silenced genomes may re-initiate the lytic gene expression program, therefore strongly arguing that viral latency is established in parallel with lytic replication at the onset of the *in vivo* infection.

### IFNβ Represses MCMV Reactivation from *In Vivo* Infected LSECs

**Figure 7:**
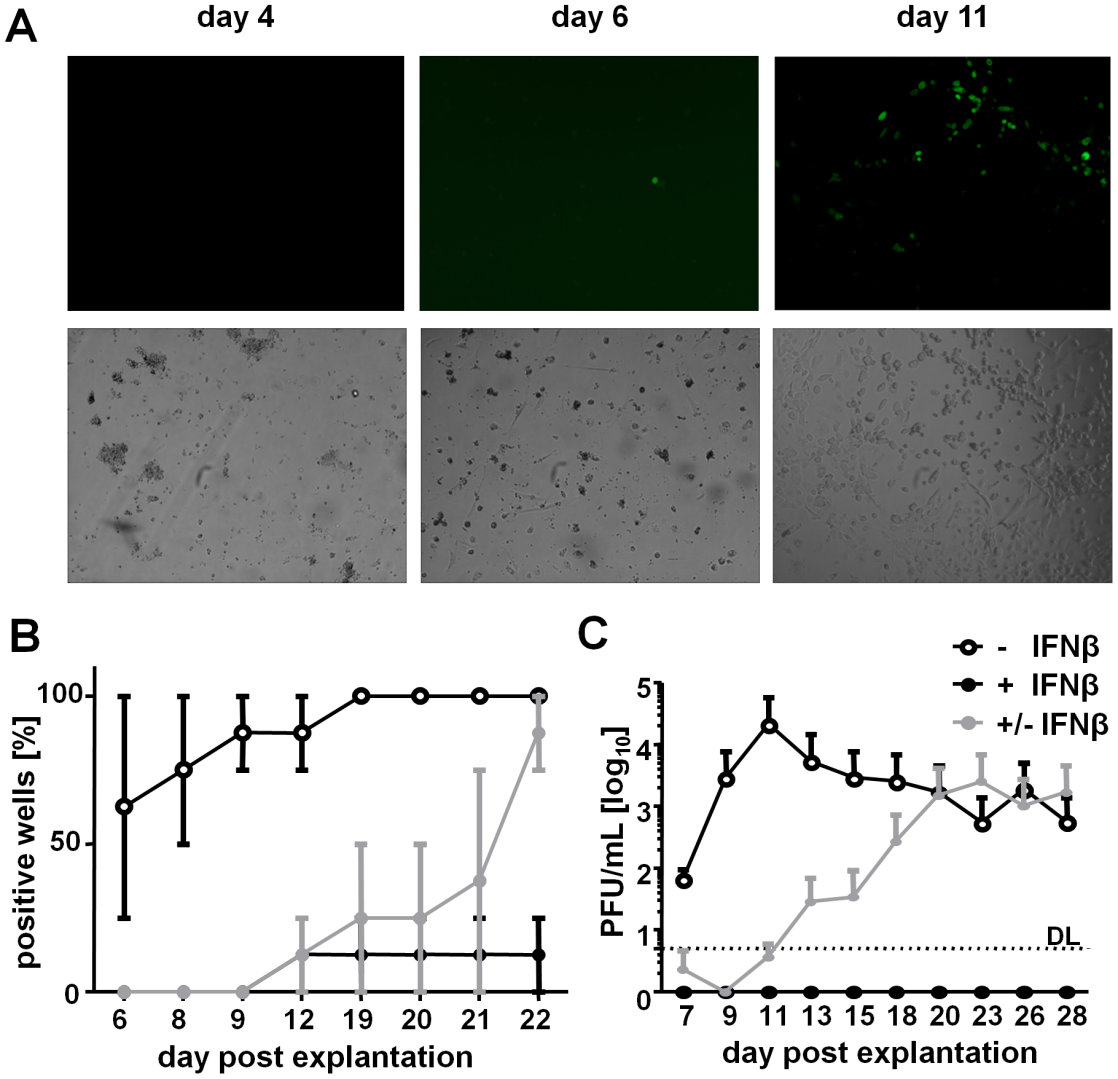
IFNβ represses MCMV reactivation from *in vivo* infected LSECs. LSECs were isolated from MCMV^r^ infected mice at 4 weeks p.i. and cultivated for up to 3 weeks. (**A**) LSECs were inspected by fluorescence microscopy for reporter gene expression. Typical microscopic pictures observed at 4, 6 and 11 days post isolation are shown. (**B**, **C**) Primary LSECs from MCMV^r^ infected mice were cultivated with 500 U/mL IFNβ (+IFNβ) or left untreated (−IFNβ). In selected wells of IFN-treated LSECs, IFNβ was removed from the cell medium at 6 days post isolation (± IFNβ). (**B**) Percentage of wells showing EYFP expression at indicated days post isolation, expressed as average ± SEM from triplicates. (**C**) SN were collected on indicated days post isolation and titrated for infectious MCMV on IFNAR^−/−^ MEFs. Graphs show average titers of triplicates (PFU/mL) ± SEM.

IFNβ reversibly silenced MCMV gene expression in LSECs infected *in vivo* and *in vitro*, a phenomenon with intriguing homologies to MCMV latency and reactivation. To determine if IFNβ would be sufficient to suppress MCMV reactivation from LSECs carrying latent viral genomes, and to define if this would also occur at the level of immediate-early gene expression, we infected mice with MCMV^r^ and isolated the LSECs from the liver of latently infected mice. Infectious MCMV^r^ is completely cleared from liver by 14 dpi [23]. Primary LSECs were isolated at 4 weeks post infection and cultivated for up to three weeks *in vitro*. Viral gene expression was monitored by fluorescence microscopy for EYFP expression. After 6 days of cultivation, the LSEC explant monolayers displayed single fluorescent cells (Figure 7A). Within a couple of days, the infection expanded resulting in numerous fluorescent cells. The majority of wells with LSECs that were cultivated in presence of IFNβ exhibited no viral gene expression (Figure 7B). In contrast, IFNβ removal at 6 days post LSEC isolation resulted in a strong increase of EYFP-positive wells, almost to levels seen in the IFN-naive controls (Figure 7B), thereby excluding the suppressive effects of IFNβ to be due to toxic effects. Finally, infectious virus shedding in the cell supernatants was confirmed only in IFN-untreated cells or upon IFNβ removal (Figure 7C). In summary, these data demonstrate that IFNβ is not only able to efficiently inhibit lytic MCMV infection following pre-treatment, but can also efficiently suppress MCMV reactivation of latently infected primary LSECs.

## Discussion

It is well-established that IFNβ inhibits lytic CMV replication, but cannot abrogate it completely [37], [38]. Recently, we reported that even high doses of IFNβ cannot activate all cells of a population, leaving a few cells unprotected [24]. We now show that this minority of cells is responsible for the failure of IFNβ to completely abrogate lytic MCMV replication. Consequently, MCMV gene expression and replication are completely blocked by IFNβ at very low doses of infection, when the probability of infection of an IFNβ-unresponsive cell is minimized. Higher doses of infectious MCMV are blocked when infecting sorted IFN-responder cells (Fig. 2). Restricting the infection to IFN-responsive cells allowed us to identify the reversible nature of the IFN-mediated inhibition of CMV replication. This could not be observed in previous studies, because viral IE gene expression in a single cell is sufficient to overcome IFNβ-induced resistance to viral replication in subsequent rounds of infection. This all-or-nothing phenotype is consistent with a model where the initial failure to contain the expression of IE1 results in a positive feedback loop, which reinforces viral transcription that can no longer be controlled by IFNβ [39], [40]. Large amounts of virus released from a single IFN-unresponsive cell are then capable of overcoming the antiviral state in the neighboring IFN-responder cells explaining the inability of IFN to fully suppress productive CMV infection in cell culture.

Consistent with this model, expression of the viral IE1 protein is crucial for the dispersion of ND10 bodies, thereby allowing transcription of viral early genes to proceed [41]. Several components of the ND10 bodies, are induced by IFN. ND10 were initially described as the nuclear domains where HCMV genomes are localized immediately upon infection [42]. Subsequent studies revealed that HCMV replication is inhibited by additive effects of ND10 components, including PML, Daxx [43] and Sp100 [44]. Daxx has been shown to be involved in chromatin modification [45], [46] and was found to bind to the MIEP of MCMV in latently infected mice [14]. In addition, a role in transcriptional suppression has been suggested for the nuclear antigen Sp100 which was shown to repress the transcriptional activity of herpes simplex virus 1 (HSV-1) promoters [47]. We showed here that IFNβ-mediated inhibition of MCMV replication critically depends on the ND10 proteins PML, Daxx and Sp100, rather than on any other IFN induced gene (Figure 5). Our study supports a critical role of ND10 bodies in limiting the viral transcription at the earliest stages of infection, and shows for the first time that this is fully reversible, and thus consistent with the molecular definition of latency. Therefore, we propose that the virus exploits the IFN-mediated induction of ND10 body components to establish latent infection in tissues strongly responding to IFN. In this context, herpesvirus latency may be understood as an immune evasion mechanism to high levels of IFN, because latency offers a choice for the virus to maintain its ability to reactivate in an environment with rampant immune responses until these responses decline.

Our data highlight a crucial role of IFNβ-mediated induction of ND10 components, similar to previous data showing the critical role of PML in the IFN repression of HSV-1 replication [48]. Our results are not necessarily limited to the establishment of latency in endothelial cells. Similar results have been recently observed in macrophages, where interferon induced ND10 expression and an MCMV refractory state at the IE expression level (M. Hassim and P. Ghazal, personal communication). Therefore, IFN may also be involved in the induction of latency in myeloid cells, and it is an intriguing possibility that this may also depend on the induction of ND10 bodies. How do the ND10 bodies silence the viral transcription? Our results may imply that the silencing is not based on the suppression of a specific promoter, but rather of any incoming episomal DNA, although this still needs to be formally confirmed. More importantly, our results showed that a complex nuclear machinery is required for MCMV silencing, because each of the shRNA knockdowns (Daxx, Sp100 or PML) were sufficient to rescue viral transcription, at least in part. Taken together, these results may imply that ND10 bodies silence viral transcription in a manner akin to programmed epigenetic control, but this hypothesis would need to be tested in a detailed study, which goes beyond the scope of this article.

IFNβ was not only able to completely inhibit lytic MCMV replication *in vitro* and *in vivo*, but also to prevent virus reactivation from latency in explant cultures. Since both IFNα and IFNβ signal through the same receptor and induce a range of similar genes, it is possible that both type I IFNs exert similar effects on MCMV latency [38], [49], [50]. On the other hand, recent evidence showed distinct differences in the downstream signaling induced by IFNβ and IFNα [51]. Furthermore IFNβ induces the secretion of IFNα in mice [52] and therefore it is possible that in our experiments the IFNβ-stimulation does not act directly, but rather by enforcing the secretion of other antiviral cytokines which may influence MCMV latency. Either way, it is unlikely that the amounts of type I IFNs which are necessary to keep the virus in check *in vitro* are produced over a prolonged time in the latently infected host, and this is also inconsistent with our kinetic monitoring of IFN responses upon MCMV infection (Fig. 6A and 6B). However, it is conceivable that individual LSECs which respond to type I IFNs generate a reservoir of latently infected cells. Once viral latency has been established, immune control may well be exerted by primed T and NK cells [53]. These cells are activated later during the infection process, but persist longer than type I IFN secreting cells and both have the potential to secrete IFNγ, and thus control lytical CMV replication [19]. MCMV specific effector T-cells are readily detectable in organs of latently infected mice [54], arguing for a strong and ongoing recruitment of immune cells to sites of virus latency, and thus for an active role of the immune system in the prevention of CMV reactivation. An additional layer of control may also result from epigenetic silencing of the viral genomes once latency has been established [10], [15]. In line with a model of epigenetic control of viral transcription, which acts on top of IFNβ mediated transcriptional suppression, IE gene expression restarted with a delay, and could only be observed approximately one week upon IFNβ retraction (Fig. 3D, 7B).

In conclusion, our study establishes a link between type I IFN signaling, ND10 bodies and reversible suppression of CMV transcription and strongly argues for their key role in the establishment of herpesviral latency.

## Materials and Methods

### Ethics Statement

All animal experiments were performed in compliance with the German animal protection law (TierSchG BGBI S. 1105; 25.05.1998). The mice were handled in accordance with good animal practice as defined by FELASA and GV-SOLAS. All animal experiments were approved by the responsible state office (Lower Saxony State Office of Consumer Protection and Food Safety) under permit number 33.9-42502-04-11/0426.

### Cells

M2-10B4 (CRL-1972; ATCC) and NIH 3T3 fibroblasts (CRL-1658; ATCC) were maintained in Dulbecco's modified Eagle medium supplemented with 10% fetal calf serum, 1% Glutamine and 1% Penicillin/Streptomycin. Primary C57BL/6, IFNAR^−/−^ and CMV-Cre MEFs were prepared and maintained as described previously [55]. Conditionally immortalized LSECs were generated and cultivated as described [23]. NIH3T3 IRF7-mCherry were generated and characterized previously [24].

### Mice

All mice were bred at the animal facility of the Helmholtz Centre for Infection Research (HZI) and maintained under specific pathogen-free conditions. Conditional deletion/reporter mice IFN-β^floxβ-luc^ and Mx2Luc reporter mice were generated and characterized previously [35], [36].

### Viruses

MCMV clones were grown on M2-10B4 cells and partially purified as described [56], with the following modification: upon ultracentrifugation, the virus pellet was resuspended in 1.2 ml of Virus standard Buffer (0.05 M Tris, 0.012 M KCl, 0.005 M EDTA) and centrifuged in a microcentrifuge for 5 minutes at 3000×g. The clear supernatant was harvested, aliquoted and stored at −80°C. The BAC-derived wild-type MCMV (MCMV WT) [57] and MCMV^r^ [23] have been described previously. The 230 kb MCMV BAC Δm157 eGFP was generated by homologous recombination of a linearized PCR fragment expressing the eGFP gene under the control of the minimal CMV promoter into the m157 genomic region of the pSM3fr BAC, essentially as described [58]. In brief, the gene was inserted by a two-step mutagenesis procedure, where in the first step the gene was introduced into the BAC, along with a kanamycin resistance gene (*kan*) flanked by frt sites, at nucleotide positions 216291 to 216874, thus replacing most of the m157 gene, including its start codon. In a subsequent step, *kan* was excised by transient expression of flip recombinase, and recombined clones were selected by kanamycin sensitivity, thus generating the Δm157/eGFP pSM3fr plasmid. The recombinant virus MCMV IE1/3^flox^ contains two *loxP* sequences which flank the open reading frames of the immediately early genes ie1 and ie3 and is a derivative of MCMV WT [57]. MCMV IE1/3^flox^ was generated by two-step recombination mutagenesis using the *galK* selection system and modified to include antibiotic resistance selection in the first mutagenesis step [59]. A linear PCR-derived recombination fragment encoding *galK* and kanamycin resistance (Kan^R^) was amplified from the pGPS/galKn plasmid [59] using primers P9 and P11 (for primer and construct sequences see supplementary table S4), inserted into SW102 E. Coli carrying the MCMV WT BAC genome and recombined BAC clones were selected on kanamycin plates. The synthetic DNA construct C1 (Geneart) was subsequently introduced, replacing the GalK/Kan gene with a loxP site at nucleotide position 177965–177974 according to the published MCMV genome annotation [60]. The second *loxP*-site was inserted with the same method, using primers P43 and P45 in the first mutagenesis step and the synthetic DNA product C2 (Eurofins MWG Operon) inserted in the second step at nucleotide position 182837–182846. The entire sequence of the final BAC clone was sequenced in an Illumina sequencer to exclude illegitimate recombination events. The newly generated BACs were transferred into MEFs and reconstituted viruses grown as described above. VSV-GFP [61] and MHV68 GFP [62] were grown as described previously.

### MCMV Growth Kinetics

Confluent monolayers of non-cycling LSECs or NIH3T3 were infected with MCMV WT or MCMV^r^ at the Multiplicity of infection (MOI) of 0.1. After 1 h, the cells were washed with PBS, supplied with fresh medium and incubated for 6 days. SN were harvested in triplicates and stored at −70°C until they were titrated on MEFs.

### IFNβ-Treatment and Virus Infections

Confluent monolayers of non-cycling LSECs were infected in 96-well plates with MCMV^r^, MHV68 GFP or VSV GFP at indicated MOIs. LSECs were treated with recombinant mouse IFNβ (PBL Interferon Source, Piscataway, NJ) as follows: (1) untreated LSECs (−IFNβ) were cultivated with normal medium throughout the experiment. (2) IFNβ-treated LSECs (+IFNβ) were stimulated 24 h before the infection and supplied with IFNβ throughout the experiment. (3) LSECs, in which the IFNβ was retracted (+/−IFNβ) were stimulated 24 h before the infection and cultivated for 7 days in the presence of IFNβ. At 7 dpi, the IFN-containing medium was exchanged with normal medium and cells were cultivated without IFNβ until the end of the experiment. For all conditions, the cells were supplied with fresh medium every 2–3 days. Infected cells were monitored by Fluorescence Microscopy for reporter gene expression at the indicated time points and wells that showed viral replication indicated by fluorescent cells, were classified as positive.

### Immunofluorescence

LSECs were cultivated on chamber slides (Thermo Scientific) and stimulated for 24 h with IFNβ. The cells were stained with Daxx (clone 25C12; 1∶25) rabbit mAb (Cell Signaling) according to the manufacture's protocol. In brief, cells were fixed with Formaldehyde, permeabilized with Triton-X-100 and anti-rabbit Alexa 488 (clone B13C; 1∶200) was used as secondary antibody. The cells were mounted with VECTASHIELD (Vector Laboratories) prior to microscopic analysis.

### *In Vivo* Infection and Reactivation Assay

6 to 10 weeks old C57BL/6 mice (Janvier) were intraperitoneally infected with 10^6^ PFU of MCMV^r^ and housed in SPF conditions throughout the experiment. Initial isolation of mouse liver non parenchymal cell (NPC) was performed according to a published protocol [63]. In brief, liver was perfused with 5 ml liver perfusion medium (Gibco-Invitrogen, Paisley, UK) and with 5 ml liver digestion medium (Gibco-Invitrogen, Paisley, UK). Upon removal of the liver from the mouse, the liver was cut in small pieces, incubated for 30 min in liver digestion medium and gently pressed through a Nylon 100 µm cell strainer (BD Falcon). Cells up from five livers were pooled, washed in PBS, resuspended in 40% Percoll (Biochrom), gently overlaid onto 70% Percoll, and centrifuged at 750× g for 20 min. NPC collected from the interface were washed twice and resuspended in PBS/1%FCS. Upon red blood cell lysis, LSECs were isolated from NPCs by immunomagnetic sorting. For this purpose, cells were counted and resuspended in 10 µl of antimouse-CD146–conjugated magnetic beads (Miltenyi Biotec) and 90 µl of PBS+1% FCS per 10^7^ nucleated cells, incubated for 15 minutes at 4°C and magnetically separated according to the manufacturer's protocol. Isolated LSECs were maintained in RPMI supplemented with 10% fetal bovine serum (FBS) (PAN Biotech, Aidenbach Germany), penicillin (100 U/mL), streptomycin (100 µg/mL), L-glutamine (2 mM), 1 mM sodium pyruvate and 0.2 mM 2-mercaptoethanol (Gibco-Invitrogen, Paisley, UK) on plates coated with 0.5% gelatin (Sigma, St. Louis, MO, USA). Cells were seeded and cultivated in an incubator at 37°C, 7% CO_2_ and 5% O_2_, at maximal humidity. LSECs were treated with recombinant mouse IFNβ and monitored by fluorescent microscopy for signs of virus reactivation as detailed above. For plaque assay, LSECs were treated with recombinant mouse IFNβ (PBL Interferon Source, Piscataway, NJ) as described above with the following modifications: LSECs were treated with 100 U/mL and the IFNβ was removed after seven days of cultivation. Triplicate SN were stored at −70°C and titrated on IFNAR^−/−^ MEFs.

### Cell Sorting

NIH3T3 IRF7-mCherry cells were stimulated with IFNβ (500 U/mL). The cells were trypsinized 24 h later, resuspended in PBS and sorted in mCherry^high^ and mCherry^low^ populations using a FACS Aria II (BD Bioscience) cell sorter.

### Stimulation and of IFNβ-Reporter Mice and *In Vivo* Imaging

For the stimulation of IFN-β^+/Δβ-luc^ mice, poly I:C (100 µg/mouse) was injected i.v. or the mice were mock injected with PBS only. To visualize the reporter gene in IFN-β^+/Δβ-luc^ and Mx2-luc mice, the mice were injected i.v. with 150 mg/kg of D-luciferin in PBS (Calipers), anesthetized using Isofluran (Baxter) and monitored using an IVIS 200 imaging system (Calipers). Photon flux was quantified using the Living Image 3.0 software (Calipers). Overlays were analyzed using the Living Image 4.1 software. Relative intensities of emitted light were presented as pseudocolor overlays ranging from red (most intense) to black (least intense). Data were expressed as radiance, quantified as photons/sec/cm^2^/sr. Steradian (sr) refers to the photons emitted from a unit of solid angular measure.

### Deep Sequencing of Nascent RNA and Transcriptome Analysis

Cells were treated with IFNβ (500 U/ml) for 24 h prior to infection. Cells were infected with WT MCMV at a nominal MOI of 1. Virus was allowed to absorb for 5 minutes at 2000 rpm in a tissue culture centrifuge and was removed immediately thereafter, which increases the infectivity rate by a factor of 10 as compared to cells infected with the same amount of virus for 1 h, in the absence of centrifugation (See Figure S5). Importantly, this increased the time resolution to the 5 minutes of virus absorption. The labeling and isolation of nascent RNA was performed for 1 h as described [29], and biological duplicates of the transcriptome (100 ng of nascent RNA per sample) were used for TruSeq RNA Library construction using TruSeq RNA sample preparation kit (Low-Throughput protocol) according to manufacturer's protocol. The final amplified library was purified using AMPure XP Beads (Agencourt). Quality of TruSeq Libraries were checked using Agilent Technologies 2100 Bioanalyzer and run on Genome Analyzer IIx (Illumina Inc.) in single end mode with length of 36 nt per read.

The program BWA [64] was used to align the reads to a reference genome composed of the mouse genome (version mm10) with the MCMV genome (NC_004065.1) inserted as an extra chromosome. Reads were read into the R statistical language version 2.15 [65] counted, and evaluated with the R package edgeR [66] following the edgeR tutorial. Annotation for the mouse was downloaded using the GenomicFeatures R package (available from the Bioconductor website) from the UCSC database, while viral annotation was created from the NCBI Genbank NC_004065 GFF file using GenomicFeatures. Mouse and virus data were analysed both separately and together, and reads per kilobase per million (RPKM) values were generated. Significantly differently expressed genes were determined by edgeR using two replicates.

### shRNA-Mediated Knockdown and Reverse Transcription PCR

shRNAs-sequences targeting murine DAXX, SP100, and PML or non-coding (NC) shRNA were generated by using the online tool from Integrated DNA Technologies (IDT) or the database from the RNAi-consortium. Design of shRNA-vector inserts for of pRNA-U6/Neo (GeneScript #SD1201) was performed according to the manufacturer's manual. Sense and antisense siRNA sequences were ordered as loop-sequences annealed before ligation into the shRNA-vector. Single clones were selected and sequenced. Both Sense and Antisense sequences of the used shRNAs are listed in Table S3. LSECs were transfected with 2 µg plasmid DNA encoding for shRNA targeting Daxx, SP100, PML or a non-targeting control-shRNA. 24 h following transfection, medium was exchanged by RPMI supplemented with IFNβ (500 U/mL), incubated for 24 h upon which the RNA was extracted from cells with TRIzol (Invitrogen), according to the manufacturers protocol. cDNA was synthesised with SyperScript II (Invitrogen) and oligo(dT)_12–18_ primers according to manufacturer's recommendation. qRT-PCR for genes of interest was performed using peqGOLD REAL-TIME (Peqlab) and SYBR Green in a LightCycler 480 (Roche) and the results were normalised to GAPDH.

## Supporting Information

Figure S1 **IFNβ reversibly blocks the replication of MHV68 and not VSV.** LSECs were infected with (**A**) 1 MOI of VSV-GFP or (**B**) 0.0001 MOI MHV68-GFP in the presence (+IFNβ, 100 or 500 U/mL) or absence of IFNß (−IFNβ). After 7 dpi, IFNβ was removed from the medium (+/− IFNβ) and wells were screened and classified as positive for GFP expression until 17 dpi. The percentages of wells showing cells with GFP expression are indicated. Graphs show the mean of three independent experiments and error bars indicate SEM.
(TIF)

Figure S2 **The loxP sites in MCMV IE1/3^flox^ are recognized by Cre recombinase.** (**A**) MEFs expressing inducible Cre recombinase (Cre.ER^T2^) were cultured for 48 h in presence of 1 µM Tamoxifen to induce it. The cells were infected with the salivary gland homogenates from MCMV IE1/3^flox^ or MCMV WT-infected mice. After additional 48 h the supernatant was harvested and used to analyse the *ie1/3* gene locus for recombination by PCR with primers P18 and P47 (see supplementary table S4) flanking the ORF of *ie1/3*. The first lane shows the negative control (N), the second lane the DNA ladder and the third lane shows the PCR product from the MCMV WT BAC (BAC). The experiment was done with samples from three different mice for each virus. (**B**) CMV-Cre and WT MEFs were infected with 0.1 MOI MCMV IE1/3^flox^, supernatants were collected from 0 to 6 dpi and titrated on MEF cells. Graph show the mean values of triplicates (± SD).
(TIF)

Figure S3 **shRNA-mediated knockdown of ND10 components.** LSECs were transfected with plasmids encoding shRNAs targeting Daxx, Sp100, PML or a non-targeting control-shRNA (ctrl). The medium was exchanged after 24 h to RPMI supplemented with 500 U/mL IFNβ where indicated. After 24 h RNA was extracted and cDNA synthesised. qRT-PCR was performed in a LightCycler 480 using SYBR Green staining. The transcripts of interest were quantified by standard dilutions with cDNA encoding vectors and normalised to GAPDH.
(TIF)

Figure S4 **Induction of IFNβ** ***in vivo*** **after poly I:C administration.** (A) Whole-body *in vivo* imaging of luciferase activity upon injection of IFNβ-reporter mice (IFN-β^+/Δβ-luc^) with poly I:C (100 µg/mouse) or PBS. The rainbow scale depicts the strength of radiance expressed as photons per second per cm^2^ per steradian (sr). Imaging was performed at 4 hours post infection (hpi). (B) Bars shows the quantification of luciferase activity by region of interest (ROI) analysis of the liver at 4 hpi from five mice and error bars indicate SD.
(TIF)

Figure S5 **Centrifugation enhances infectivity of MCMV.** LSECs were infected with 0.1 MOI MCMV^r^ using centrifugal enhancement of 5 min at 800 g or incubated for 1 h at 37°C. The medium was exchanged after the centrifugation or the 1 h-incubation and replaced with normal culture medium. Histograms show the percentage of EYFP-expressing cells as analyzed by flow cytometry after 3 dpi.
(TIF)

Table S1 **Regulation of viral genes in IFNβ-treated LSECs.** Normalised counts of MCMV genes expressed in non-treated (− IFN) and treated (+ IFN) LSECs (stimulated with 500 U/mL IFN for 24 h) after 1 hpi. The presented numbers represent RPKM (Reads per kilo base per million), to normalise the reads for the length of the gene.
(XLSX)

Table S2 **Regulation of cellular genes in IFNβ-treated LSECs.** Normalised counts of cellular genes expressed in non-treated (− IFN) and treated (+ IFN) LSECs (stimulated with 500 U/mL IFN for 24 h). The presented numbers represent RPKM (Reads per kilo base per million), to normalise the reads for the length of the gene.
(XLSX)

Table S3 **List of shRNA sequences.** The sense and antisense sequences that were used for the shRNA-expressing plasmids (described in detail in Material and Methods).
(XLSX)

Table S4 **Primer and construct sequences.** The sequences of the primers and constructs that were used to generate the recombinant MCMV IE1/3^flox^ (Generation is described in detail in Material and Methods).
(XLSX)
