# Supplementary figures and images for: Reversible Silencing of Cytomegalovirus Genomes by Type I Interferon Governs Virus Latency
Source: PLoS Pathog. 2014 Feb 20;10(2):e1003962. doi: 10.1371/journal.ppat.1003962 (PMC3930589; doi:10.1371/journal.ppat.1003962)

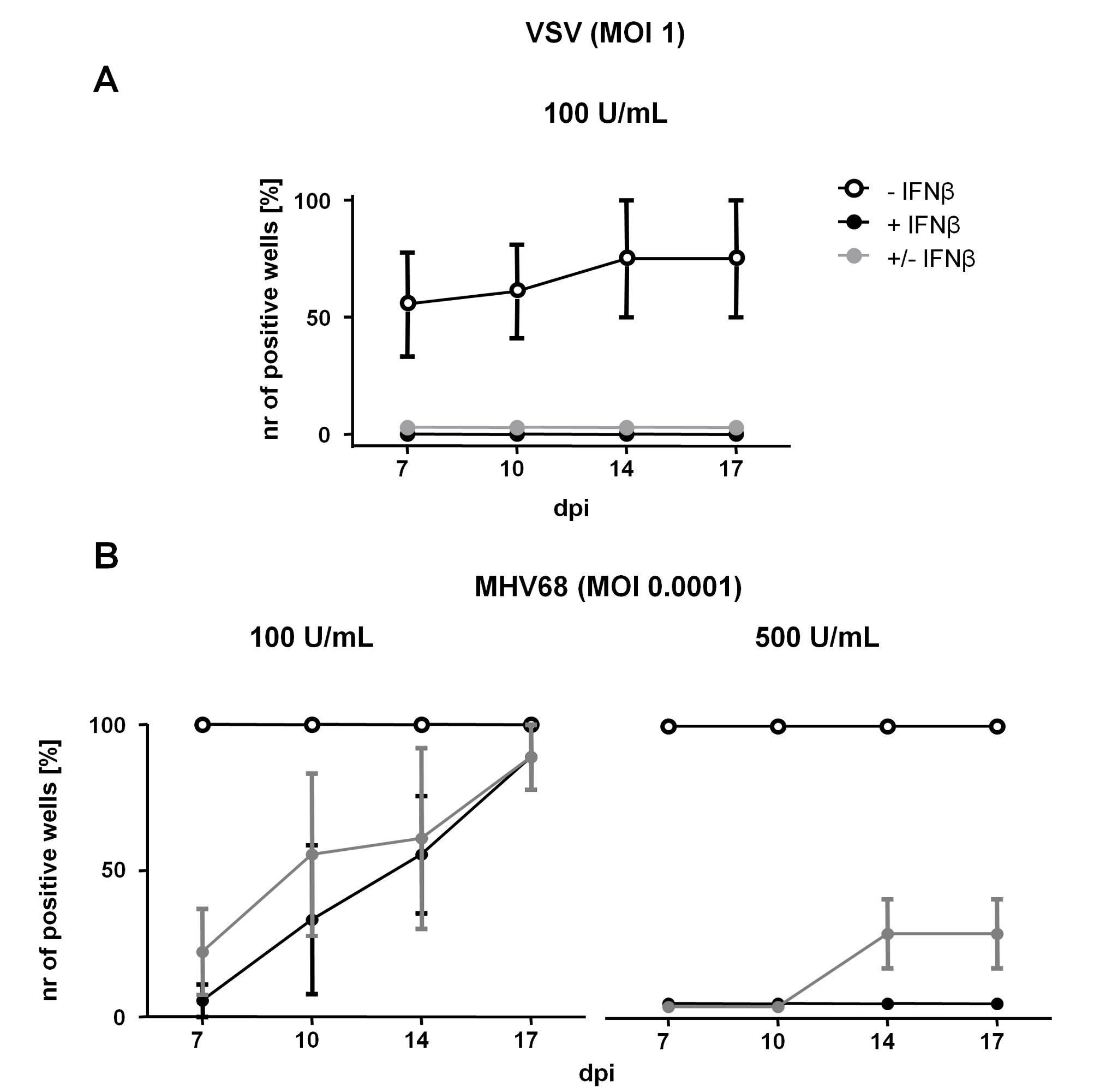

Supplement: Figure S1 — IFNβ reversibly blocks the replication of MHV68 and not VSV. LSECs were infected with (A) 1 MOI of VSV-GFP or (B) 0.0001 MOI MHV68-GFP in the presence (+IFNβ, 100 or 500 U/mL) or absence of IFNß (−IFNβ). After 7 dpi, IFNβ was removed from the medium (+/− IFNβ) and wells were screened and classified as positive for GFP expression until 17 dpi. The percentages of wells showing cells with GFP expression are indicated. Graphs show the mean of three independent experiments and error bars indicate SEM. (TIF) [file ppat.1003962.s001.tif]

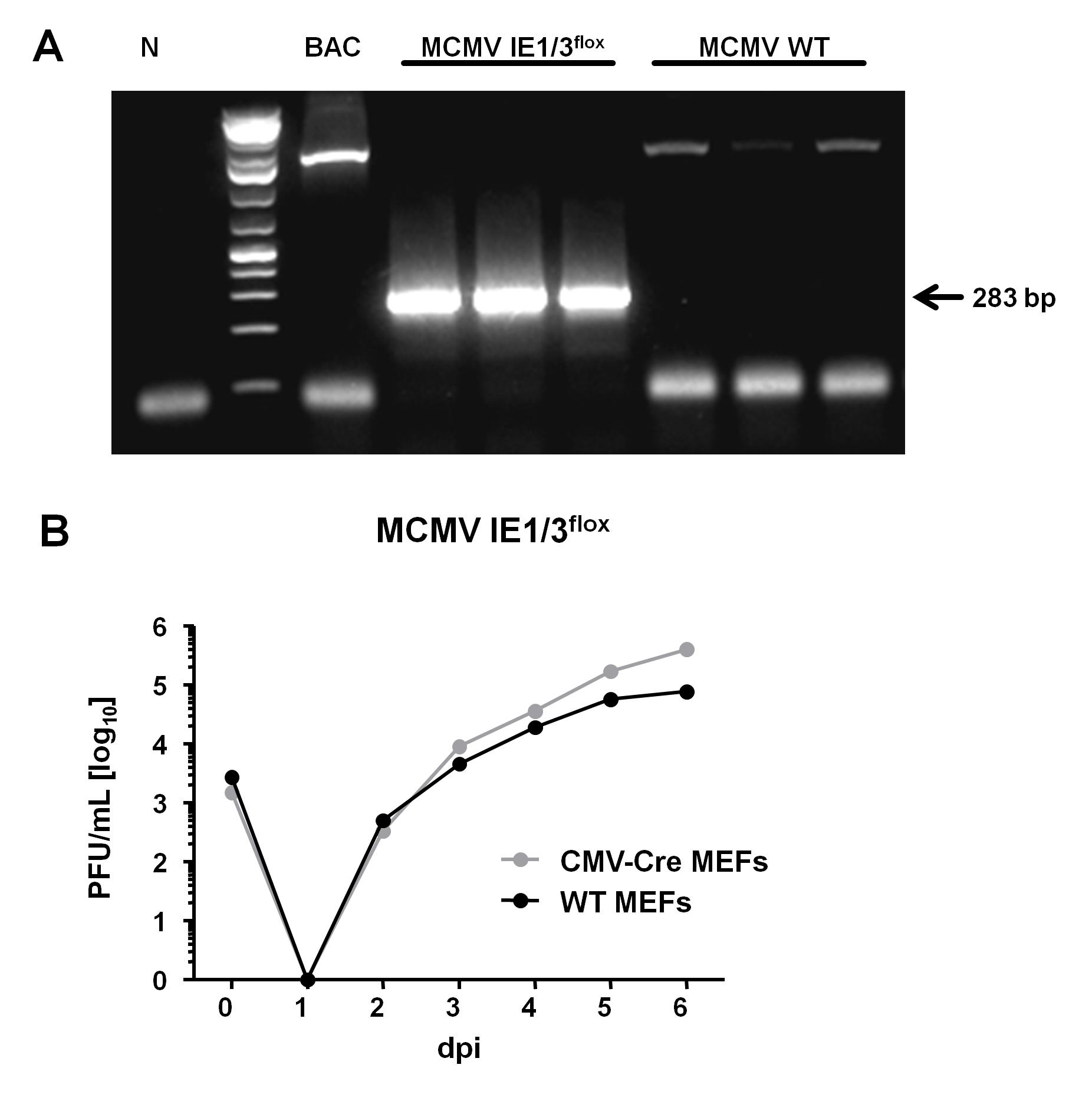

Supplement: Figure S2 — The loxP sites in MCMV IE1/3flox are recognized by Cre recombinase. (A) MEFs expressing inducible Cre recombinase (Cre.ERT2) were cultured for 48 h in presence of 1 µM Tamoxifen to induce it. The cells were infected with the salivary gland homogenates from MCMV IE1/3flox or MCMV WT-infected mice. After additional 48 h the supernatant was harvested and used to analyse the ie1/3 gene locus for recombination by PCR with primers P18 and P47 (see supplementary table S4) flanking the ORF of ie1/3. The first lane shows the negative control (N), the second lane the DNA ladder and the third lane shows the PCR product from the MCMV WT BAC (BAC). The experiment was done with samples from three different mice for each virus. (B) CMV-Cre and WT MEFs were infected with 0.1 MOI MCMV IE1/3flox, supernatants were collected from 0 to 6 dpi and titrated on MEF cells. Graph show the mean values of triplicates (± SD). (TIF) [file ppat.1003962.s002.tif]

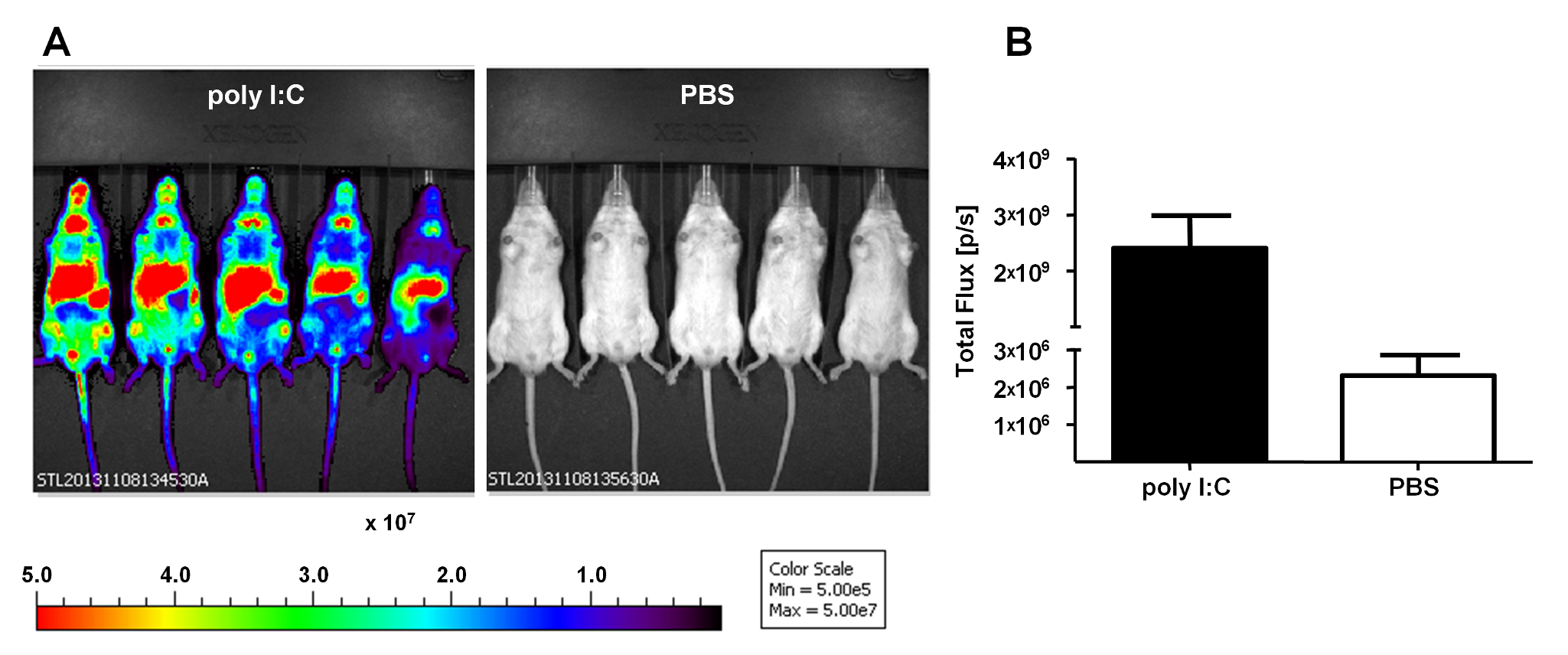

Supplement: Figure S4 — Induction of IFNβ in vivo after poly I:C administration. (A) Whole-body in vivo imaging of luciferase activity upon injection of IFNβ-reporter mice (IFN-β+/Δβ-luc) with poly I:C (100 µg/mouse) or PBS. The rainbow scale depicts the strength of radiance expressed as photons per second per cm2 per steradian (sr). Imaging was performed at 4 hours post infection (hpi). (B) Bars shows the quantification of luciferase activity by region of interest (ROI) analysis of the liver at 4 hpi from five mice and error bars indicate SD. (TIF) [file ppat.1003962.s004.tif]
